# Supplementary figures and images for: A comprehensive assessment of multi-system responses to a renal inoculation of uropathogenic E. coli in swine
Source: PLoS One. 2020 Dec 11;15(12):e0243577. doi: 10.1371/journal.pone.0243577 (PMC7732124; doi:10.1371/journal.pone.0243577)

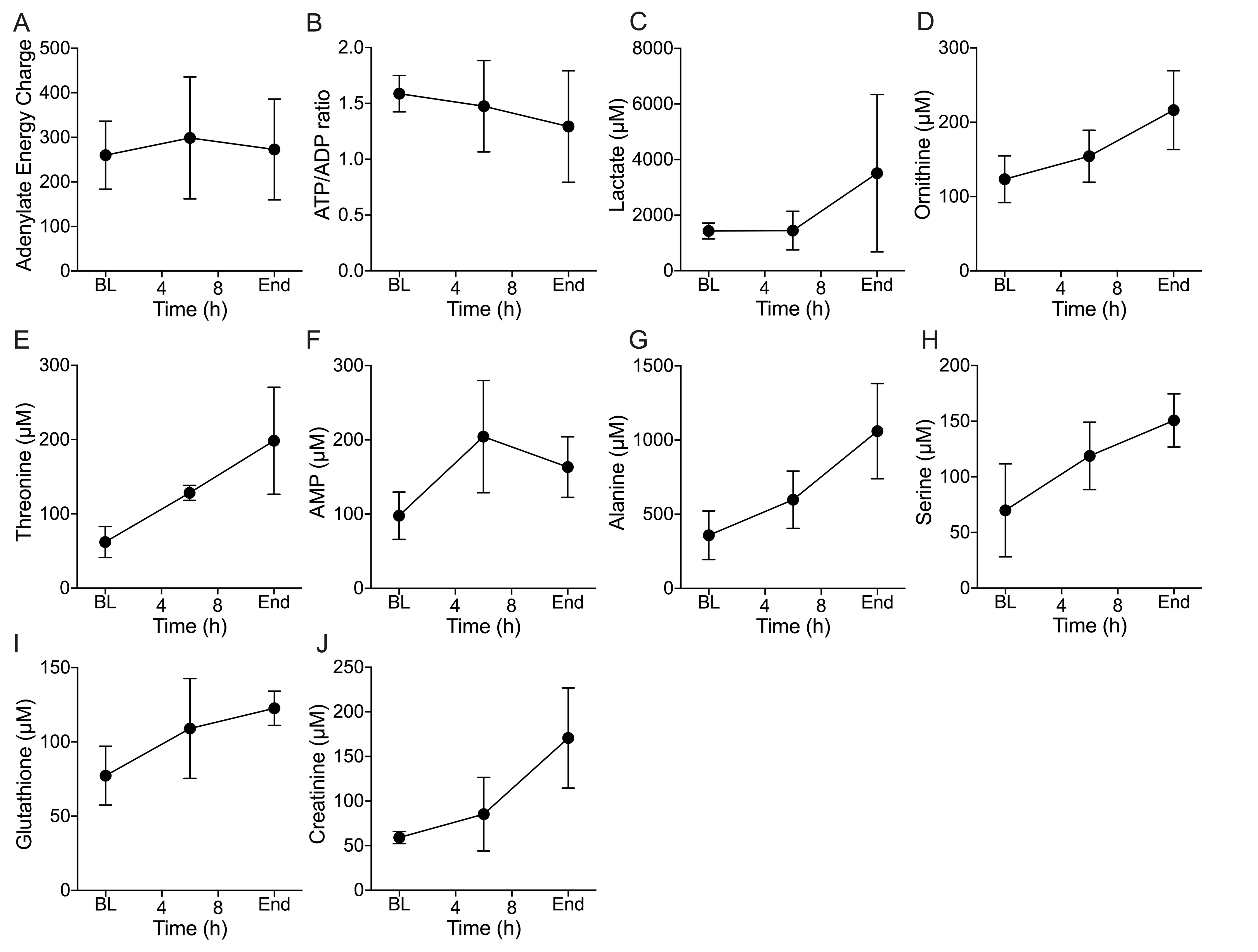

Supplement: S1 Fig — The adenylate energy charge (A) did not change, but the ATP/ADP ratio (B) trended downward. Lactate, as measured by NMR (C), followed the same trend as the clinical measurement and was not significantly changed during the course of the experiment (ANOVA false discovery rate [FDR] corrected p-value = 0.3). In addition to the nine metabolites with FDR < 10%, there were an additional seven metabolites with FDR of < 15%. These were (D) ornithine (FDR = 13%), (E) threonine (FDR = 13%), (F) AMP (FDR = 13%), (G) alanine (FDR = 13%), (H) serine (FDR = 13%), (I) glutathione (FDR = 15%), and (J) creatinine (FDR = 15%). Data are the mean (SE) from four animals. BL = baseline. (TIF) [file pone.0243577.s001.tif]

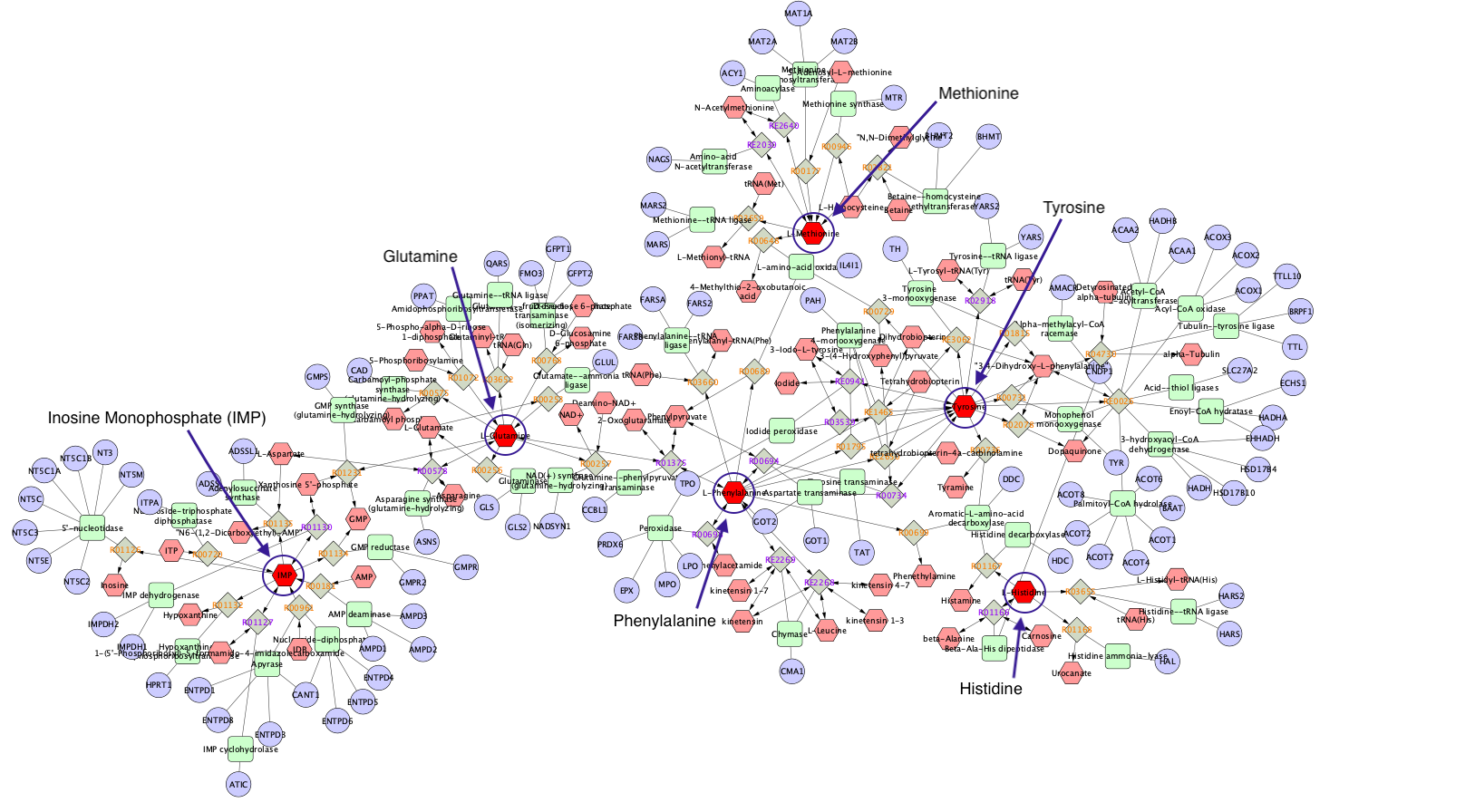

Supplement: S2 Fig — Of the nine metabolites with an FDR-corrected ANOVA p value of < 0.10, six (inosine monophosphate, glutamine, methionine, tyrosine, phenylalanine and histidine) mapped into a single network. The figure was generated by uploading the Kyoto Encyclopedia of Genes and Genomes (KEGG) identification (ID) numbers of the nine metabolites into Metscape (3.1.3 metscape.ncibi.org/), a plugin application for Cytoscape (3.8.0 cytoscape.org). The human library was used. Metabolites are designated by red hexagons with dark red being those that were manually entered into Metscape. The gray squares represent reactions, green round-corner squares, enzymes and blue circles, genes. (TIF) [file pone.0243577.s002.tif]

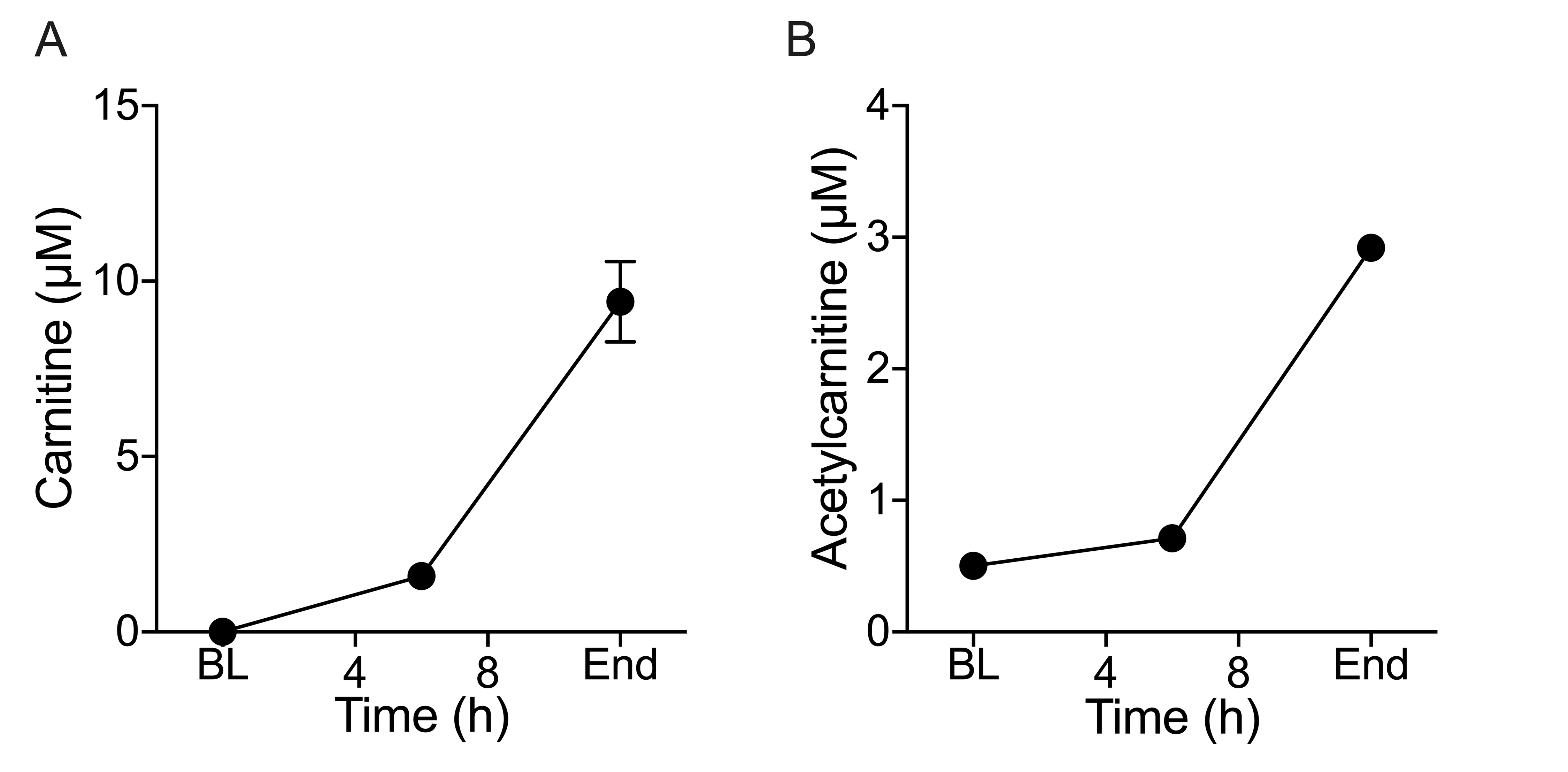

Supplement: S3 Fig — Concentrations of (A) carnitine were not detectable in any samples at baseline (BL), only one sample had a detectable concentration at 6h but carnitine was detected in all samples by the end of the experiment. Acetylcarnitine (B) was only detectable on one sample at BL and 6h and only two samples at the end of the experiment. These metabolites were removed from the primary analysis because of data missingness but are shown here because they have previously been shown to be important as indicators of sepsis severity (see online supplement text). Data are the mean (SE, when applicable) from four animals. (TIF) [file pone.0243577.s003.tif]

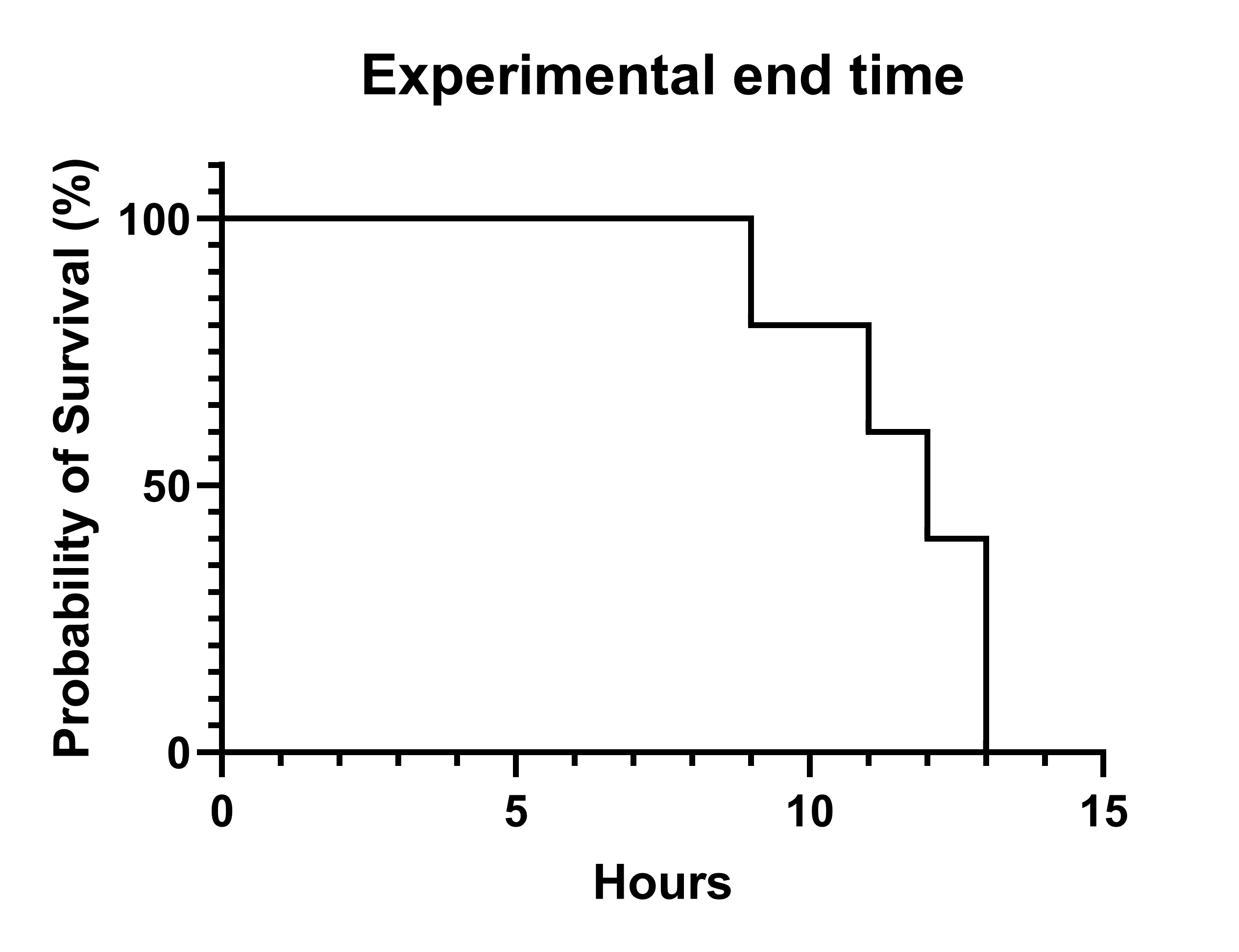

Supplement: S4 Fig — Plot of survival times for each animal used in the study. (TIF) [file pone.0243577.s004.tif]
