# Supplementary material for: A comprehensive assessment of multi-system responses to a renal inoculation of uropathogenic E. coli in swine
Source: PLoS One. 2020 Dec 11;15(12):e0243577. doi: 10.1371/journal.pone.0243577 (PMC7732124; doi:10.1371/journal.pone.0243577)
Supplement: S1 Table — Hemodynamic and biochemical parameters were measured in each animal at baseline (pre-infection) and at the termination of the experiment. These included core temperature, heart rate, mean arterial pressure, central venous pressure (CVP); pulmonary artery pressure (PAP); creatinine, blood urea nitrogen (BUN); glucose, lactate, total bilirubin, alanine transaminase (ALT); platelets, hemoglobin, hematocrit, white blood cells (WBC; with percent neutrophils and lymphocytes); arterial pH; bicarbonate (HCO3-); end tidal carbon dioxide pressure (PetCO2); oxygen consumption, partial pressure of oxygen (PaO2); fraction of inspired oxygen (FiO2); and central venous oxygen saturation (ScvO2). (DOCX) [file pone.0243577.s005.docx]

| **S1** **Table.** Baseline and end of experiment parameters for all animals. | | | | | | |
| --- | --- | --- | --- | --- | --- | --- |
|  | **Baseline**  **(n=5)** | |  | **End of Experiment**  **(n=5)** | | |
|  | **Mean** | **SD** |  | **Mean** | **SD** | **Tukey p value** |
| Core Temperature (°C) | 37.9 | 0.80 |  | 43.0 | 1.23 | 0.005 |
| Heart Rate (BPM) | 74.2 | 5.89 |  | 142 | 43.24 | 0.13 |
| Mean Arterial Pressure (mmHg) | 85.5 | 9.31 |  | 27.6 | 5.03 | 0.05 |
| CVP (mmHg) | 7.7 | 1.0 |  | 4.7 | 0.5 | 0.03 |
| PAP (mmHg) | 21.3 | 7.4 |  | 24.2 | 4.6 | 0.98 |
| Creatinine (mg/dL) | 1.36 | 0.11 |  | 3.59 | 0.99 | 0.02 |
| BUN (mg/dL) | 7.4 | 3.44 |  | 21.4 | 6.54 | 0.002 |
| Glucose (mmol/L) | 8.5 | 1.4 |  | 3.7 | 1.2 | 0.04 |
| Lactate (mEq/L) | 1.5 | 0.93 |  | 8.0 | 3.80 | 0.09 |
| Total Bilirubin (µmol/L) | 5.1 | 1.2 |  | 7.7 | 6.0 | 0.87 |
| ALT (U/L) | 47.2 | 16.8 |  | 49.0 | 11.0 | 0.5 |
| Platelets (10^9^/L) | 279.6 | 102.36 |  | 203.0 | 88.41 | 0.2 |
| Hemoglobin (g/dL) | 10.8 | 1.6 |  | 14.6 | 1.8 | 0.10 |
| Hematocrit (%) | 41 | 6.6 |  | 54 | 3.6 | 0.05 |
| WBC (10^9^/L) | 17.0 | 3.59 |  | 18.4 | 8.05 | 0.4 |
| Neutrophil (%) | 37.5 | 7.81 |  | 74.4 | 7.95 | 0.01 |
| Lymphocyte (%) | 61.58 | 7.60 |  | 21.7 | 6.91 | 0.004 |
| Arterial pH | 7.5 | 0.02 |  | 7.3 | 0.16 | 0.52 |
| HCO_3_^-^ | 29.5 | 2.2 |  | 18.3 | 3.5 | 0.02 |
| PetCO_2_ | 44 | 3.9 |  | 53 | 21.5 | 0.93 |
| Oxygen consumption (mL/kg/min) | 3.9 | 2.5 |  | 3.2 | 3.1 | 1.0 |
| PaO_2_/FiO_2_ ratio | 456.6 | 62.11 |  | 315.2 | 118.22 | 0.34 |
| ScvO_2_ (%) | 59 | 2 |  | 17 | 10 | 0.005 |

CVP (central venous pressure); PAP (pulmonary artery pressure); BUN (Blood Urea Nitrogen); ALT (alanine transaminase); AST (aspartate transaminase); WBC (White Blood Cells); PetCO_2_ (end tidal carbon dioxide pressure); PaO_2_ (Partial Pressure of Oxygen); FiO_2_ (Fraction of Inspired Oxygen); ScvO_2_ (Central Venous Oxygen Saturation).
