# Supplementary material for: A comprehensive assessment of multi-system responses to a renal inoculation of uropathogenic E. coli in swine
Source: PLoS One. 2020 Dec 11;15(12):e0243577. doi: 10.1371/journal.pone.0243577 (PMC7732124; doi:10.1371/journal.pone.0243577)
Supplement: S2 Table — (DOCX) [file pone.0243577.s006.docx]

| **Metabolite Name** | **KEGG ID** |
| --- | --- |
| 2-Aminoadipate | C00956 |
| 2-Hydroxybutyrate | C05984 |
| 2-Oxoisocaproate | C00233 |
| Acetylcarnitine | C02571 |
| ADP | C00008 |
| AMP | C00020 |
| ATP | C00002 |
| Alanine | C00041 |
| Betaine | C00719 |
| Carnitine | C00318 |
| Choline | C00114 |
| Creatine | C00300 |
| Creatinine | C00791 |
| Dimethyl sulfone | C11142 |
| Glucose | C00221 |
| Glutamate | C00025 |
| Glutamine | C00064 |
| Glutathione | C00051 |
| Glycine | C00037 |
| Hippurate | C01586 |
| Histidine | C00135 |
| Hypoxanthine | C00262 |
| IMP | C00130 |
| Isoleucine | C00407 |
| Lactate | C00186 |
| Leucine | C00123 |
| Lysine | C00047 |
| Malonate | C00383 |
| Methionine | C00073 |
| Ornithine | C00077 |
| Phenylalanine | C00079 |
| Proline | C00148 |
| Pyruvate | C00022 |
| Serine | C00065 |
| Taurine | C00245 |
| Threonine | C00188 |
| Trimethylamine N-oxide | C01104 |
| Tyrosine | C00082 |
| Valine | C00183 |

### S2 Table. ^1^H-Nuclear Magnetic Resonance (NMR)-detected and quantified swine whole blood metabolites with Kyoto Encyclopedia of Genes and Genome (KEGG) Identifications (ID)
