# Supplementary material for: A comprehensive assessment of multi-system responses to a renal inoculation of uropathogenic E. coli in swine
Source: PLoS One. 2020 Dec 11;15(12):e0243577. doi: 10.1371/journal.pone.0243577 (PMC7732124; doi:10.1371/journal.pone.0243577)
Supplement: S1 File — (DOCX) [file pone.0243577.s007.docx]

**Online Supporting Information**

A comprehensive assessment of multi-system responses to a renal inoculation of

uropathogenic *E. coli* in swine

Mohamad Hakam Tiba, Brendan M. McCracken, Robert P. Dickson, Jean A. Nemzek, Carmen I. Colmenero, Danielle C. Leander, Thomas L. Flott, Rodney C. Daniels, Kristine E. Konopka, J. Scott VanEpps, Kathleen A. Stringer and Kevin R. Ward

Quantitative ^1^H-nuclear magnetic resonance (NMR) Metabolomics

**Materials and Methods**

*Sample Collection*

Whole blood (WB) samples were collected from the indwelling line in the internal jugular vein. Blood was collected into 4ml Vacutainer brand® tubes containing sodium heparin (cat # 367871). Following collection, tubes were inverted several times to ensure adequate mixing and were immediately placed on ice. Each of two 600uL aliquots were added to screw-top cryogenic storage tubes (2mL; Wheaton cat # W985867) and flash frozen in liquid nitrogen. Frozen samples were placed on dry ice until the completion of the experiment at which time they were transferred in liquid nitrogen and stored until the time of assay. The remaining whole blood volume in the collection tube was centrifuged (1300 x *g* for 10 min at 4°C). Aliquots of plasma (~600uL) were transferred screw-top cryogenic storage tubes (2mL), flash frozen in liquid nitrogen, placed on dry ice, and stored in liquid nitrogen for future assays.

*Preparation of Samples for Nuclear Magnetic Resonance (NMR) Spectroscopy*

Deuterium oxide (99.8 atom%D) and chloroform (ACS reagent grade) were obtained from Acros Organics (Pittsburgh, PA, USA). Methanol (NF, absolute), monobasic sodium phosphate (monohydrate), and dibasic sodium phosphate (heptahydrate) were purchased from Fisher Scientific (Pittsburgh, PA, USA). Deuterium chloride, and sodium deuteroxide were obtained from Sigma Aldrich (St. Louis, MO, USA). DSS-d_6_(4,4-dimethyl-4-silapentane-1-sulfonic acid) internal standard with 0.2% sodium azide was purchased from Chenomx, Inc. (IS-2) (Edmonton, AB, Canada).

At the time of assay, samples were thawed on ice and subjected to a methanol-chloroform precipitation as previously described [1]. Briefly, samples were thawed in an ice-water bath after which 500 µL of blood was transferred to a microcentrifuge tube and 1ml of a 1:1 methanol-chloroform solution was added. Samples were sonicated for 2 minutes at 4°C, then incubated at -20°C for 20 minutes, and then centrifuged (13,400 x *g* at 4°C for 30 minutes). The aqueous supernatant was transferred to a new microcentrifuge tube and dried by lyophilization. Samples were then resuspended in 600 µL of 50mM sodium phosphate buffer in D_2_O for NMR analysis.

*Acquisition 1D-^1^H NMR Spectra and Analysis*

1D-^1^H NMR spectra were acquired and analyzed as previously described [1]. Briefly, sample volume was measured and recorded before the addition of 50 µL of 4.99 mM DSS-d_6_ with 0.2% sodium azide following which sample pH was measured to between 6.5 and 7.5 and if necessary, corrected by the addition of 20 µL of 0.1 mM of either deuterium chloride or sodium deuteroxide. Samples were then transferred to 5 mm 500 MHz precision NMR tubes (Wilmad Lab Glass, Vineland, NJ).

Spectra were acquired at the University of Michigan’s Biochemical NMR Core Laboratory on a Varian (now Agilent, Inc.) 11.74 Tesla (500 MHz) NMR spectrometer equipped with a 5-mm Agilent “One-probe,” using a VNMRS console operated by host software VNMRJ 4.0. Spectra were recorded using 32 scans of the first increment of a 1 H, 1 H-NOESY pulse sequence, at a room temperature of 295.45 ± 0.3 K.

Spectra were analyzed using the Chenomx NMR Suite 8.2 program. Phase shifting, baseline correction, and water removal in each spectrum was done using the Processor module, after which compounds were identified and quantified using the Profiler module as previously described [1]. The concentration data was then scaled to correct for differences in initial sample volume. A total of 39 metabolites were named and quantified (Table S2). The entire data set can be found on the NIH Metabolomics Workbench (<https://www.metabolomicsworkbench.org/>) under the accession # PR000953.

*Statistical Analysis*

Metabolomics data were pre-processed using Metaboanalyst [2]. First, metabolites missing more than 30% of concentration values were removed from the data set; this reduced the data set to 33 metabolites. Prior to statistical analysis, remaining missing values were replaced with half of the minimum concentration value present in the original dataset. Data were then log transformed and range scaled. The concentration data across the three time points were compared using a repeated measures one-way ANOVA followed by a post-hoc Tukey HSD test when applicable. Analysis of metabolomics data was conducted in PRISM (PRISM 8, 2019). ANOVA p values were corrected for false discovery rate (FDR) using the method of Storey, et al [3]. Metabolites were ranked by ascending FDR and the Kyoto Encyclopedia of Genes and Genomes (KEGG) identification (ID) of those with an FDR of < 10% were uploaded into Cytoscape and network maps were created using Metscape [4].

**Results**

Table S1 shows the baseline and end of experiment parameters presented in the main manuscript with associated ANOVA *post hoc* (Tukey) p values. The ^1^H-NMR detected and quantified metabolites and their respective Kyoto Encyclopedia of Genes and Genomes (KEGG) identification numbers are shown in Table S2. The time course plots of metabolites with an FDR-corrected ANOVA p value < 0.15 are shown in Fig S1. The Metscape generated network of metabolites with an FDR-corrected ANOVA p value < 0.10 is shown in Fig S2. Notably, in this model of progressive systemic infection, acetylcarnitine and carnitine, which we and others have previously shown to be indicative of sepsis severity [5, 6], were not detectable in all samples until the end of the experiment (Fig S3).

**References**

1. McHugh CE, Flott TL, Schooff CR, Smiley Z, Puskarich MA, Myers DD, et al. Rapid, Reproducible, Quantifiable NMR Metabolomics: Methanol and Methanol: Chloroform Precipitation for Removal of Macromolecules in Serum and Whole Blood. Metabolites. 2018;8(4). Epub 2018/12/19. doi: 10.3390/metabo8040093. PubMed PMID: 30558115; PubMed Central PMCID: PMCPMC6316042.

2. Chong J, Wishart DS, Xia J. Using MetaboAnalyst 4.0 for Comprehensive and Integrative Metabolomics Data Analysis. Current Protocols in Bioinformatics. 2019;68(1):e86. doi: 10.1002/cpbi.86.

3. Storey JD. A direct approach to false discovery rates. Journal of the Royal Statistical Society Series B-Statistical Methodology. 2002;64:479-98. doi: Unsp 1369-7412/02/64479

Doi 10.1111/1467-9868.00346. PubMed PMID: WOS:000177425500009.

4. Karnovsky A, Weymouth T, Hull T, Tarcea VG, Scardoni G, Laudanna C, et al. Metscape 2 bioinformatics tool for the analysis and visualization of metabolomics and gene expression data. Bioinformatics. 2012;28(3):373-80. Epub 2011/12/03. doi: 10.1093/bioinformatics/btr661. PubMed PMID: 22135418; PubMed Central PMCID: PMCPMC3268237.

5. Chung KP, Chen GY, Chuang TY, Huang YT, Chang HT, Chen YF, et al. Increased Plasma Acetylcarnitine in Sepsis Is Associated With Multiple Organ Dysfunction and Mortality: A Multicenter Cohort Study. Crit Care Med. 2019;47(2):210-8. Epub 2018/11/01. doi: 10.1097/ccm.0000000000003517. PubMed PMID: 30379669.

6. Puskarich MA, Finkel MA, Karnovsky A, Jones AE, Trexel J, Harris BN, et al. Pharmacometabolomics of l-carnitine treatment response phenotypes in patients with septic shock. Ann Am Thorac Soc. 2015;12(1):46-56. Epub 2014/12/17. doi: 10.1513/AnnalsATS.201409-415OC. PubMed PMID: 25496487; PubMed Central PMCID: PMCPMC4342803.
