# Supplementary material for: A comprehensive assessment of multi-system responses to a renal inoculation of uropathogenic E. coli in swine
Source: PLoS One. 2020 Dec 11;15(12):e0243577. doi: 10.1371/journal.pone.0243577 (PMC7732124; doi:10.1371/journal.pone.0243577)
Supplement: Attachment — Submitted filename: Response to Reviewers.docx [file pone.0243577.s010.docx]

**Comments to the Author**

1. If the authors have adequately addressed your comments raised in a previous round of review and you feel that this manuscript is now acceptable for publication, you may indicate that here to bypass the “Comments to the Author” section, enter your conflict of interest statement in the “Confidential to Editor” section, and submit your "Accept" recommendation.

**Reviewer #1: (No Response)**

2. Is the manuscript technically sound, and do the data support the conclusions?
The manuscript must describe a technically sound piece of scientific research with data that supports the conclusions. Experiments must have been conducted rigorously, with appropriate controls, replication, and sample sizes. The conclusions must be drawn appropriately based on the data presented.

**Reviewer #1: Yes**

3. Has the statistical analysis been performed appropriately and rigorously?

**Reviewer #1: Yes**

4. Have the authors made all data underlying the findings in their manuscript fully available?
The [PLOS Data policy](http://www.plosone.org/static/policies.action#sharing) requires authors to make all data underlying the findings described in their manuscript fully available without restriction, with rare exception (please refer to the Data Availability Statement in the manuscript PDF file). The data should be provided as part of the manuscript or its supporting information, or deposited to a public repository. For example, in addition to summary statistics, the data points behind means, medians and variance measures should be available. If there are restrictions on publicly sharing data—e.g. participant privacy or use of data from a third party—those must be specified.

**Reviewer #1: Yes**

5. Is the manuscript presented in an intelligible fashion and written in standard English?
PLOS ONE does not copyedit accepted manuscripts, so the language in submitted articles must be clear, correct, and unambiguous. Any typographical or grammatical errors should be corrected at revision, so please note any specific errors here.

**Reviewer #1: Yes**

6. Review Comments to the Author
Please use the space provided to explain your answers to the questions above. You may also include additional comments for the author, including concerns about dual publication, research ethics, or publication ethics. (Please upload your review as an attachment if it exceeds 20,000 characters)

**Reviewer #1:**

**Comment:** The manuscript has been improved and the changes have largely answered the concerns that were expressed. However, the suggestion that this approach is novel deserves a supportive discussion. A paragraph in the discussion based upon what is already known about porcine models is needed to put the current work into context.

It is necessary that the manuscript make it explicit why this models is more representative of sepsis than previously published studies using pigs. Perhaps taking advantage of the reviews that the author as mentioned, the discussion could add a paragraph to put the work into better context

**Response:** We thank the reviewer for this comment. We have added a paragraph to highlight the novelty of our model and what sets it apart from other models. In addition to our model’s faithfulness to the clinical course of severe infection, the model’s longitudinal observation of temporal events, its breadth and magnitude of physiologic, metabolic, and organ specific data collected, sets it apart from murine or other large animal models of sepsis. These features make our model distinct from other porcine models of sepsis particularly since, to date, most have induced sepsis via peritonitis or by direct intravenous infusion of either a pathogen or endotoxin. Furthermore, many have employed interventions such as fluid supplementation, antibiotics or vasopressors which mask the natural course of illness. We also added citation [47] which lists the MQTiPPS review Part II recommendations. Within other recommendations, it advocates for the discontinuation of endotoxin challenge use as well as modeling of the sepsis syndromes at sites other than the peritoneal cavity (e.g., lung, urinary tract, brain). Our model aligns closely with these recommendations.

The manuscript has been updated to address the reviewer’s comment and few other formatting issues (redlined manuscript: lines 423-426, 432-437, 671-679, and 685-687).
